# AHNAK and Inflammatory Markers Predict Poor Survival in Laryngeal Carcinoma

**DOI:** 10.1371/journal.pone.0056420

**Published:** 2013-02-07

**Authors:** Claudia A. Dumitru, Agnes Bankfalvi, Xiang Gu, Reinhard Zeidler, Sven Brandau, Stephan Lang

**Affiliations:** 1 Department of Otorhinolaryngology, University of Duisburg-Essen, Essen, Germany; 2 Department of Pathology/Neuropathology, University of Duisburg-Essen, Essen, Germany; 3 Department of Otorhinolaryngology, Ludwig Maximilians University, Munich, Germany; Virginia Commonwealth University, United States of America

## Abstract

AHNAK/Desmoyokin is a giant protein which has been recently linked to reorganization of the actin cytoskeleton, cellular migration and invasion. Here, we investigated the role of AHNAK in the pathophysiology of larynx carcinoma-one of the major subtypes of head and neck cancer. To this end, we analysed AHNAK expression in tumor tissues from 83 larynx carcinoma patients in relation to overall survival. We found that tumoral AHNAK overexpression significantly associated with poor survival of these patients both in univariate and multivariate analysis. In further studies, we combined the prognostic value of AHNAK with selected markers of inflammation, such as macrophage migration inhibitory factor (MIF) and tumor-infiltrating neutrophils (CD66b-positive cells). Both MIF and neutrophils have been linked to enhanced tumoral migration and poor clinical outcome in patients with orohypopharynx carcinoma-another major subtype of head and neck cancer. Interestingly, we found that synchronous high levels of AHNAK and MIF or AHNAK and neutrophils, respectively, were stronger predictors of poor survival than AHNAK alone. Synchronous high levels of all three markers were the strongest predictors of poor survival in our patient cohort. Taken together, our findings propose novel strategies for an accurate prognosis in larynx carcinoma and suggest potential mechanisms of inflammation-mediated tumor progression.

## Introduction

Head and neck cancer (HNC) is the eighth most common type of cancer worldwide. Despite multiple and aggressive therapeutic interventions, there has been no fundamental improvement in the 5-year survival rates of the patients over the past decades [1], [2]. Therefore, there is an urgent need to: i) identify clinicopathological factors for accurate diagnosis, prognosis and therapeutic prediction in these patients and to ii) understand the biology and molecular mechanisms behind the respective factors.

AHNAK (desmoyokin) is a protein of exceptionally large size (700 kDa) that is expressed in a variety of cell types [3]. Early studies proposed that AHNAK localized mainly in the nuclei-a potential consequence of the nuclear localization sequences (NLSs) present in the carboxy-terminal domain of the protein [4]. Additionally, it was proposed that AHNAK might associate with the Golgi network [4], although later studies did not observe co-localization of AHNAK with Golgi markers such as mannosidase II or tgn38 [5]. Other studies reported that AHNAK localized in the cytoplasm and/or associated with the plasma membrane [6], [7]. The apparent discrepancy regarding the localization of AHNAK might be explained by the ability of this protein to shuttle between various subcellular compartments. For instance, it has been shown that AHNAK can translocate from the cytoplasm to the plasma membrane of keratinocytes in a manner dependent on Ca^2+^ and Protein Kinase C [7]. Furthermore, AHNAK was shown to contain a nuclear export signal (NES) sequence which ‘allowed’ it to be excluded from the nuclei of epithelial cells following cell-cell contact and activation of Protein Kinase B, respectively [8]. At functional level, AHNAK was shown to be involved in various cellular processes, including calcium regulation and organization of the actin cytoskeleton [9], [10]. In tumor cells, AHNAK was recently found to be essential for pseudopodia formation and tumoral migration/invasion [11]. Other recent studies proposed that the AHNAK gene might be involved in mutagenic transformation of colon epithelial cells and, thus, carcinogenesis [12]. A potential role of AHNAK as prognostic marker for survival in cancer patients has, however, never been shown thus far.

Presently, it is well established that solid tumors display an inflammatory microenvironment characterized by large numbers of tumor-infiltrating immune cells [13]. Within this microenvironment, the immune cells of the host are 'reprogrammed' by the tumor cells to acquire pro-tumoral activities. Although less characterized than tumor-associated macrophages (TAMs) or tumor-infiltrating lymphocytes (TILs), tumor-infiltrating neutrophils are emerging as important players in the pathophysiology of cancer. Within the tumor tissue, neutrophils can modulate several cellular processes which may ultimately lead to tumor progression. Neutrophils were shown to modulate angiogenesis in several murine tumor models [14], [15], [16] and were recently associated with angiogenesis progression in hepatocellular carcinoma patients [17]. Further studies showed that neutrophils could directly modulate the biology and functions of tumor cells by promoting their migration, invasion or proliferation (reviewed in [18]). Thus, it is not surprising that very recent studies reported an association of high numbers of tumor-infiltrating neutrophils with advanced disease and poor clinical outcome in patients with different types of cancer, such as renal cancer, hepatocellular cancer, non-small-cell lung carcinoma (NSCLC) or melanoma (reviewed in [19]). Recently, we demonstrated that high neutrophilic infiltration of the tumor tissue associated with high tumor (T) stage and poor survival in head and neck (orohypopharynx) cancer patients with advanced disease [20]. Furthermore, our *in vitro* studies indicated a direct interaction between neutrophils and head and neck cancer cells by showing that neutrophils were ‘primed’ by the tumor cells to release pro-inflammatory factors which promoted tumoral migration in a feed-back manner [21], [22].

The exact molecular mechanisms responsible for tumor-host interactions in head and neck and other types of cancer have been only partially clarified. Selected soluble inflammatory mediators, such as cytokines, chemokines and metabolites of the arachidonic acid pathway, have been found to change the function and differentiation of immune cells [23]. Among these molecules, macrophage migration inhibitory factor (MIF) is emerging as an important regulator of inflammation in cancer [24]. A number of studies found that high levels of MIF in the tumor tissues or serum of patients with different types of cancer associated with advanced disease and poor clinical outcome (reviewed in [25]). Recent studies from our group demonstrated that overexpression of tumoral MIF associated with poor overall survival in patients with orohypopharyngeal cancer [21]. More importantly, we identified MIF as one of the 'missing links' in the tumor-neutrophil interraction and showed that head and neck cancer cells released MIF which, subsequently, enhanced the proinflammatory functions of neutrophils to promote tumoral migration [21].

This study investigated the relevance of AHNAK, MIF and tumor-infiltrating neutrophils (CD66b-positive cells) for the survival of patients with laryngeal carcinoma-a major subtype of head and neck cancer. We demonstrated that AHNAK overexpression significantly associated with poor survival in these patients. Interestingly, we found that high levels of AHNAK together with high MIF expression or high neutrophilic infiltration, respectively, were stronger associated with poor survival than AHNAK alone. Synchronous high levels of MIF and tumor-infiltrating neutrophils had stronger predictor values over the individual markers as well. Finally, patients with high levels of all three markers displayed the shortest survival in the entire patient cohort. Thus, our study proposes novel strategies for a more accurate prognosis in larynx carcinoma and suggests potential mechanisms of inflammation-mediated tumor progression.

## Materials and Methods

### Study subjects

Tissue microarrays (TMAs) were obtained from 83 patients with head and neck squamous cell carcinoma of the larynx. The patients were treated at the Department of Otorhinolaryngology (University of Duisburg-Essen) between 1995 and 2002, and clinical follow-up was retrieved. Patient characteristics are summarized in Table 1. Healthy epithelial tissues were obtained from tonsils or palatine uvulas following tonsillectomy or uvulopalatopharyngoplasty (UPPP), respectively. All studies were approved by the ethics committee of the University Hospital Essen (nr. 12-5192-BO). The data were analysed anonymously and the ethics committee provided a waiver of the need for informed consent.

**Table 1:** Characteristics of the patients used for analysis of AHNAK, MIF and CD66b, indicating number, gender, TNM stage, histological grading and AJCC stage.

		patients per group	
Patient characteristics		number	% of total
	all patients	83	100
	gender		
	male	78	94.0
	female	5	6.0
	T- stage		
	T1	14	16.9
	T2	26	31.3
	T3	25	30.1
	T4	18	21.7
	N- stage		
	N0	54	65.1
	N1	10	12.0
	N2a	0	0
	N2b	10	12.0
	N2c	9	10.8
	N3	0	0
**distant metastasis**			
	M0	83	100
	M1	0	0
**differentiation grade**			
	grade 1	6	7.2
	grade 2	64	77.1
	grade 3	13	15.7
	AJCC- stage		
	stage I	12	14.5
	stage II	21	25.3
	stage II	22	26.5
	stage IV	28	33.7

### Antibodies and reagents

Polyclonal rabbit anti-human AHNAK antibodies (catalog number HPA019070) were obtained from Sigma (St. Louis, MO, USA). Monoclonal mouse anti-CD66b antibodies clone 80H3 (catalog number IM0166) were from Immunotech (Marseille Cedex, France). Monoclonal mouse anti-MIF antibodies (catalog number MAB289) were obtained from R&D Systems (Abingdon, United Kingdom).

### Tissue microarrays: construction and immunohistochemical analysis

Tissue cores with a diameter of 3 mm were extracted from formalin-fixed/paraffin-embedded tumor tissue blocks using a skin biopsy punch (PFM, Cologne, Germany). The tissue cores were then brought into recipient blocks and cut into 5 µM sections. Antigen retrieval was performed by HIER (Heat-Induced Epitope Retrieval) in citrate buffer pH 6.0. Samples were stained with 0.6 µg/ml anti-AHNAK antibodies using an automated staining device (Dako Autostainer; DakoCytomation, Hamburg, Germany). Secondary and tertiary immunoreactions were performed with a commercially available anti-rabbit IgG detection kit (En-Vision; DakoCytomation, Hamburg, Germany). Colorimetric reactions were developed with diamino-benzidine (DAB). Staining of TMAs with anti-MIF and anti-CD66b antibodies, respectively, has been described previously [20], [21]. Analysis was performed with a Zeiss Axioscope 2 microscope at 200×(AHNAK and MIF) and 400×(CD66b) magnification, respectively. Blinded scoring was performed independently by authors C.A.D., X.G. and A.B. (senior histopathologist).

### AMIDA screening

Tumors elicit an immune response, leading to the generation of antibodies specific for tumoral antigens. AMIDA technology (autoantibody-mediated identification of antigens) identifies proteins preferentially recognized by antibodies from cancer patients over those from healthy controls. Here, we used AMIDA to screen for tumor-associated antigens in 4 head and neck cancer patients versus 4 healthy donors, as previously described [26]. Briefly, plasma antibodies were isolated using Protein G Montage spin columns (Millipore, Billerica, USA). Purified antibodies were covalently coupled to protein G beads (Amersham Biosciences, Freiburg, Germany) and incubated with cell lysates from FaDu cells. Bound proteins were eluted into 2D lysis buffer (9 M urea, 4% v/v Chaps, 2.5 mM EDTA). Isoelectric focusing was done on IPGphor system using pH 4–7 IPG strips (GE Healthcare) and proteins were separated on a vertical 12% PAGE in an Ettan Dalt II unit (GE Healthcare, Freiburg, Germany). Differentially precipitated protein spots were excised and analyzed. Mass spectrometry was performed at the Protein Analysis Unit at the Ludwig-Maximilians-University of Munich.

### Statistical analysis

Clinical data were analysed with the SPSS statistical software version 18.0 (SPSS Inc, Chicago, IL, USA). Survival curves (5-years cutoff) were plotted according to the Kaplan-Meier method. Significance was initially tested in univariate analysis using the log-rank test or univariate Cox regression. Multivariate analysis was used to determine the independent prognostic value of selected variables using Cox's proportional hazards linear regression model. In all studies, the level of significance was set at *p*≤0.05.

## Results

### AHNAK, MIF and CD66b in larynx carcinoma: expression and scoring system

**Figure 1:**
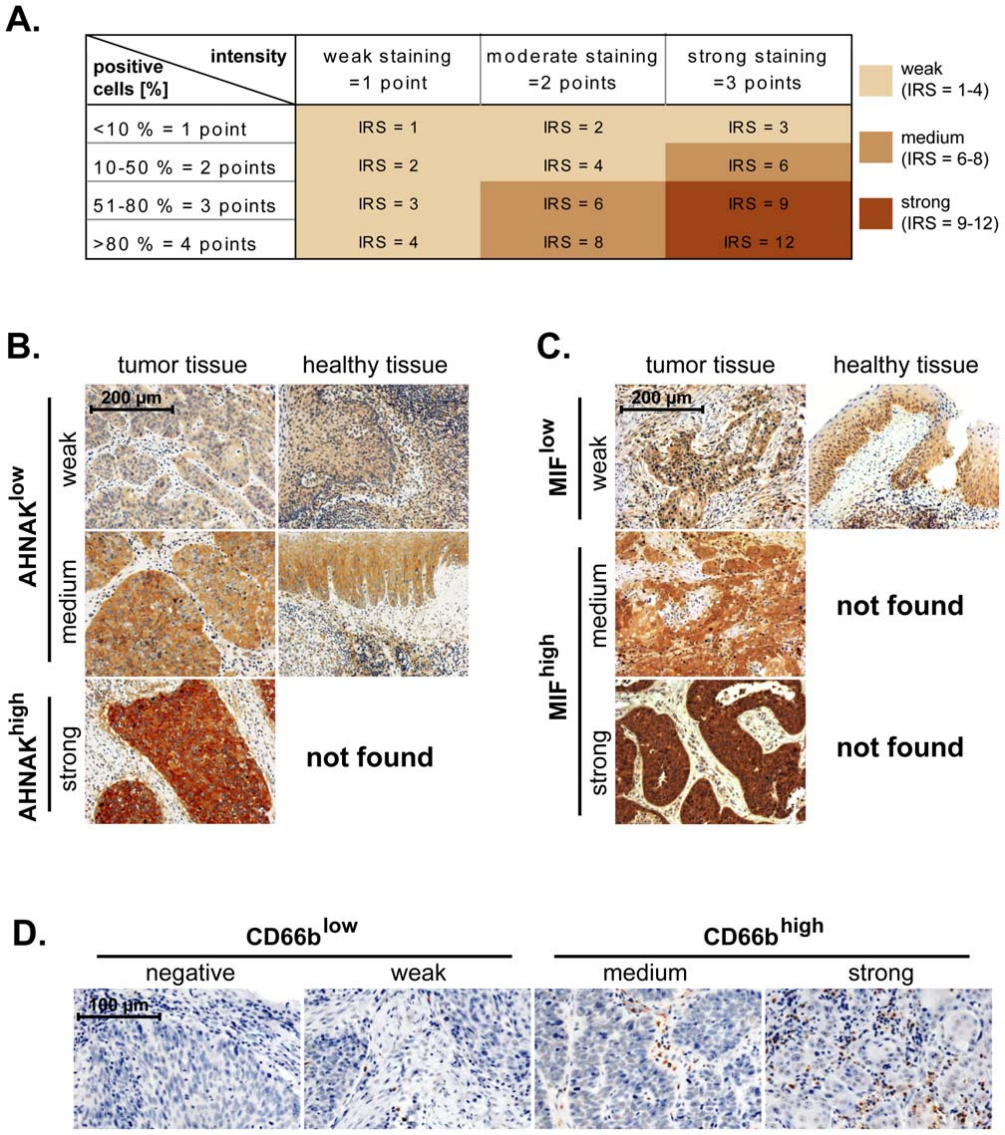
AHNAK, MIF and CD66b expression and scoring in larynx carcinoma tissues. (**A**) Malignant laryngeal and healthy epithelial tissues were stained against AHNAK and MIF. The immunoreactive score (IRS) was calculated as intensity of the staining reaction multiplied by the percentage of positive cells. Based on the IRS values, AHNAK and MIF were scored as *weak*, *medium* and *strong*. (**B**) Representative micrographs indicating that *strong* levels of AHNAK are found only in malignant tissues, while healthy epithelial tissues displayed either *weak* or *medium* levels of AHNAK. Consequently, the weak and medium AHNAK-expressing specimens were considered as ‘AHNAK^low^’ while strong AHNAK-expressing specimens were termed ‘AHNAK^high^’. (**C**) Representative micrographs indicating that *medium* and *strong* levels of MIF are found only in malignant tissues, while healthy epithelial tissues displayed *weak* levels of MIF. Consequently, the weak MIF-expressing specimens were considered as ‘MIF^low^’ while medium and strong MIF-expressing specimens were termed ‘MIF^high^’. (**D**) Representative micrographs showing *negative*, *weak*, *medium* and *strong* infiltration of larynx carcinoma tissues by neutrophils (CD66b-positive cells). Negative and weak samples were considered as CD66b^low^ while medium and strong samples as CD66b^high^. Scale bars are indicated in the upper-left corner of each figure and apply for all panels of the respective figure.

Using AMIDA technology we initially screened for novel tumor-associated antigens (TAAs) in sera from head and neck cancer patients and we identified AHNAK as a potential candidate (see Materials and Methods section). Next, we determined the expression of AHNAK at protein level by immunohistochemical analysis of tissue microarrays (TMAs) from 83 larynx carcinoma patients. As control, AHNAK expression was determined in healthy epithelial tissues from tonsil (n = 9) and palatine uvula (n = 3). AHNAK expression was scored as intensity of staining multiplied by the percentage of positive cells (IRS = immunoreactive score) (Figure 1A). An IRS of 1–4 was considered as *weak* AHNAK, IRS 6–8 as *medium* and IRS 9–12 represented *strong* AHNAK levels (Figure 1A). In healthy epithelial tissues we observed *weak* and *medium* levels of AHNAK, but no *strong* AHNAK expression (Figure 1B). Consequently, we considered that *weak* and *medium* AHNAK represent basal levels of the protein (from here on termed ‘AHNAK^low^’), while *strong* AHNAK represents overexpressed levels of the protein (from here on termed ‘AHNAK^high^’).

In another set of studies we stained TMAs from the same patients as above against selected markers of inflammation. We chose MIF and CD66b (neutrophil marker) because they have been shown to associate with advanced disease and poor survival in orohypopharynx carcinoma patients-another major subtype of head and neck cancer [20], [21]. Expression of MIF, likewise that of AHNAK, was initially scored based on the IRS system (see above). However, in healthy epithelial tissues we only found MIF to be weakly expressed (Figure 1C). Consequently, we considered that *weak* MIF represents basal levels of the protein (from here on termed ‘MIF^low^’), while *medium* and *strong* MIF represent overexpressed levels of the protein (from here on termed ‘MIF^high^’). The levels of tumor-infiltrating neutrophils (CD66b-positive cells) were initially assessed as *negative*, *weak*, *medium* and *strong* (Figure 1D). For the final statistical analysis the *negative* and *weak* CD66b samples were considered as CD66b^low^, while the *medium* and *strong* samples were considered as CD66b^high^, as previously published [20].

### AHNAK, MIF and CD66b in larynx carcinoma: univariate analysis of survival

**Figure 2:**
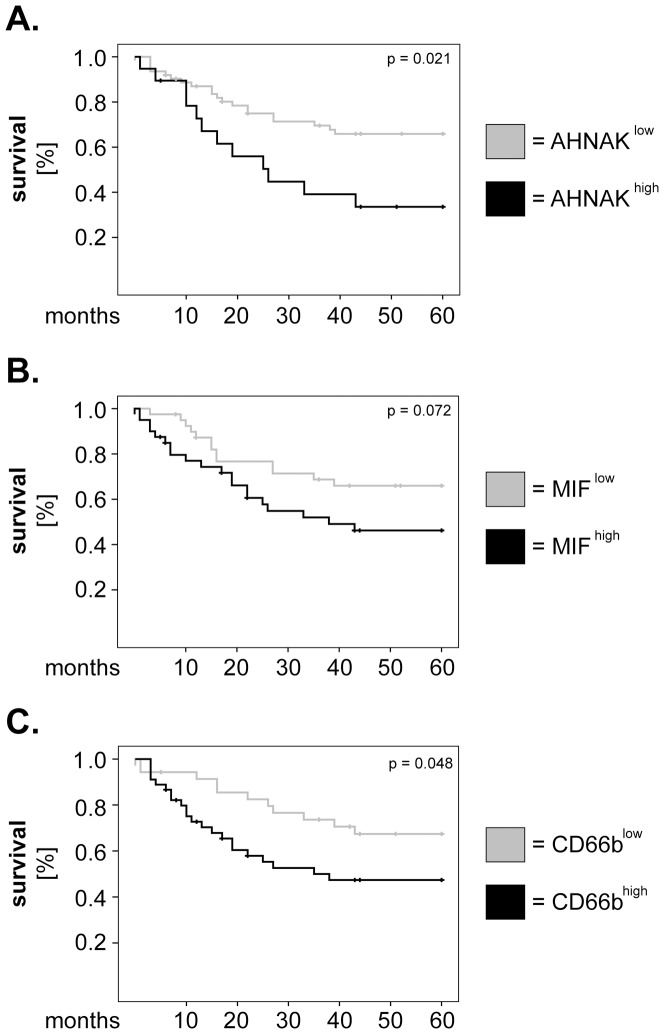
Univariate analysis of survival for AHNAK, MIF and tumor-infiltrating neutrophils in larynx carcinoma patients. Kaplan-Meier 5-years survival curves were plotted for patients with low versus high levels of (**A**) AHNAK, (**B**) MIF and (**C**) tumor-infiltrating neutrophils (CD66b). Statistical testing was performed with the log-rank test.

In further studies, we determined whether there might be a relationship between expression levels of AHNAK, MIF or CD66b and 5-years overall survival of larynx carcinoma patients. To this end, survival curves were plotted according to the Kaplan-Meier method and significance was tested by log-rank test. The results showed that patients with high levels of AHNAK (AHNAK^high^) or CD66b (CD66b^high^) had significantly shorter survival than AHNAK^low^ or CD66b^low^ patients (p = 0.021 and p = 0.048, respectively; log-rank) (Figures 2A and 2C). Patients with high levels of MIF (MIF^high^) tended to have worse survival than those with MIF^low^ (p = 0.072; log-rank) (Figure 2B); however a larger cohort of patients might be needed to reach statistical significance for MIF.

**Figure 3:**
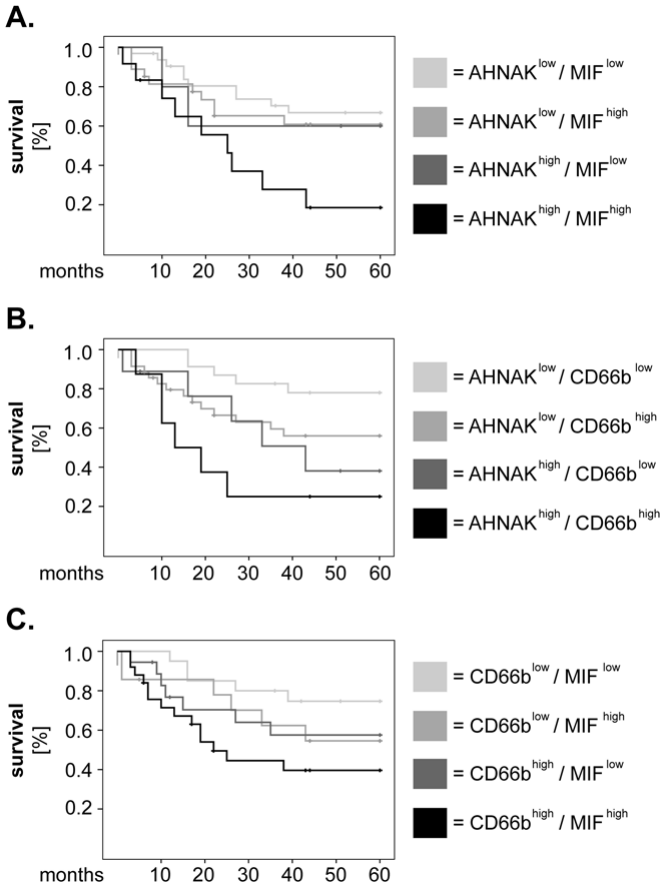
Combined analysis of AHNAK, MIF and tumor-infiltrating neutrophils regarding survival of larynx carcinoma patients. Larynx carcinoma patients were divided into 4 groups for each combination of markers and survival curves were plotted according to the Kaplan-Meier method. (**A**) combined analysis of AHNAK and MIF, (**B**) combined analysis of AHNAK and CD66b and (**C**) combined analysis of CD66b and MIF. Statistical analysis was performed by univariate Cox regression and the results are summarized in Table 2.

Next, we investigated the relevance of AHNAK, MIF and CD66b taken in combination regarding survival of larynx carcinoma patients. To this end, we divided our cohort of patients into 4 groups for each combination of markers (i.e. AHNAK^low^/MIF^low^, AHNAK^low^/MIF^high^, AHNAK^high^/MIF^low^ and AHNAK^high^/MIF^high^). Similar groups were built for AHNAK/CD66b and for CD66b/MIF, respectively. We plotted Kaplan-Meier survival curves for the above-mentioned marker combinations (**Figure 3A–C**) and observed that patients with 'double-high' phenotype had consistently shorter overall survival than their counterparts (**Figure 3A–C**). To test the significance of these results, we performed univariate Cox regression analysis for overall survival. The groups with ‘double-low' phenotype were considered as the dummy variable (value = 1). As indicated in Table 2, patients with synchronous high levels of AHNAK/MIF (HR = 3.48, 95% CI = 1.40–8.61, p = 0.007), AHNAK/CD66b (HR = 5.96, 95% CI = 1.80–19.78, p = 0.003) and CD66b/MIF (HR = 3.41, 95% CI = 1.22–9.53, p = 0.019) had significantly higher hazard of death than the other groups of patients (Table 2).

**Table 2:** Univariate Cox regression analysis for survival in patients with different combinations of AHNAK/MIF, AHNAK/CD66b and MIF/CD66b.

		Hazard ratio for survival			patients per group	
Cox regression univariate		HR	95% CI	p-value	number	% of total
AHNAK	MIF					
low	low	1			32	42.1
low	high	1.34	0.55–3.23	0.509	27	35.5
high	low	1.37	0.30–6.27	0.681	5	6.57
high	high	3.48	1.40–8.61	0.007	12	15.7
AHNAK	CD66b					
low	low	1			23	30.6
low	high	2.47	0.89–6.88	0.083	35	46.6
high	low	3.38	0.98–11.72	0.054	9	12
high	high	5.96	1.80–19.78	0.003	8	10.6
CD66b	MIF					
low	low	1			20	25.9
low	high	2.06	0.63–6.77	0.232	14	18.1
high	low	1.99	0.63–6.28	0.237	18	23.3
high	high	3.41	1.22–9.53	0.019	25	32.4

HR = hazard ratio; 95% CI = 95% confidence interval. Significant values are indicated by asterisks (*).

### AHNAK, MIF and CD66b in larynx carcinoma: multivariate analysis of survival

**Figure 4:**
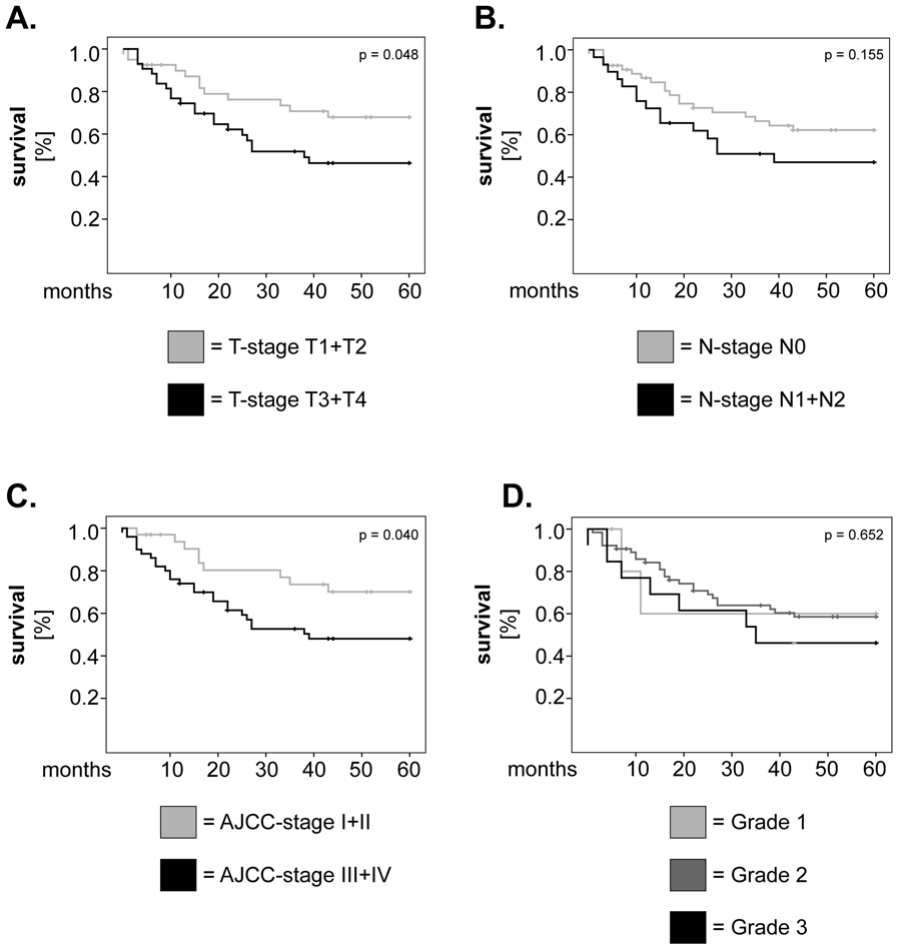
Univariate analysis of survival for other potentially-relevant clinical parameters in larynx carcinoma patients. Kaplan-Meier 5-years survival curves were plotted for patients with (**A**) low versus high T-stage, (**B**) presence or absence of lymph node metastasis, (**C**) low versus high AJCC stage and (**D**) well-, moderately- and poorly-differentiated tumors. Statistical testing was performed with the log-rank test.

In further studies we sought to confirm the relative values of AHNAK, MIF and CD66b as prognostic markers in larynx carcinoma by multivariate survival analysis using Cox regression model. To build this model, we tested what other factors might influence survival in our patient cohort and, consequently, plotted Kaplan-Meier curves for tumor stage (T-stage), lymph node metastasis (N-stage), AJCC stage and histological grading (Figure 4). We found that T-stage (Figure 4A) as well as AJCC stage (Figure 4C) were significantly associated with overall survival (p = 0.048 and p = 0.040, respectively; log-rank). Next, we adjusted our multivariate model for T- and AJCC-stage and tested the significance of AHNAK, MIF and CD66b as single parameters or in combination. The results showed that, when analysed individually, AHNAK remained a strong and significant prognostic marker (HR = 3.17, 95% CI = 1.45–6.94, p = 0.004) (Table 3). Similarly, patients with synchronous high levels of AHNAK/MIF (HR = 4.40, 95% CI = 1.71–11.30, p = 0.002), AHNAK/CD66b (HR = 8.17, 95% CI = 2.26–29.45, p = 0.001) and CD66b/MIF (HR = 3.55, 95% CI = 1.21–10.40, p = 0.021) continued to exhibit significantly higher hazard of death compared to their counterparts (Table 3).

**Table 3:** Multivariate Cox regression analysis for AHNAK, MIF and CD66b taken individually or in combination.

			Hazard ratio for survival			patients per group	
	Cox regression multivariate		HR	95% CI	p-value	number	% of total
		AHNAK					
		low	1			59	72.8
		high	3.17	1.45–6.94	0.004	22	27.2
		MIF					
		low	1			40	50.0
		high	1.76	0.87–3.56	0.113	40	50.0
		CD66b					
		low	1			35	43.7
		high	1.98	0.93–4.18	0.073	45	56.3
	AHNAK	MIF					
	low	low	1			32	42.1
	low	high	1.23	0.51–2.97	0.639	27	35.5
	high	low	1.73	0.37–8.06	0.484	5	6.57
	high	high	4.40	1.71–11.30	0.002	12	15.7
	AHNAK	CD66b					
	low	low	1			23	30.6
	low	high	2.83	0.97–8.22	0.083	35	46.6
	high	low	5.13	1.36–19.27	0.015	9	12.0
	high	high	8.17	2.26–29.45	0.001	8	10.6
	CD66b	MIF					
	low	low	1			20	25.9
	low	high	2.19	0.65–7.32	0.203	14	18.1
	high	low	2.47	0.75–8.13	0.135	18	23.3
	high	high	3.55	1.21–10.40	0.021	25	32.4
AHNAK	CD66b	MIF					
low	low	low	1			15	16.9
low	high	low	3.28	0.77–13.97	0.107	17	23.3
low	low	high	1.82	0.29–11.28	0.520	8	11.0
low	high	high	4.00	0.98–16.35	0.053	18	24.7
high	low	low	2.13	0.21–20.78	0.514	3	4.1
high	high	low	7.09	1.48–34.03	0.014	6	8.2
high	low	high	4.84	0.48–48.42	0.180	2	2.7
high	high	high	13.80	2.98–63.87	0.001	5	6.8

HR = hazard ratio; 95% CI = 95% confidence interval. Significant values are indicated by (*).

**Figure 5:**
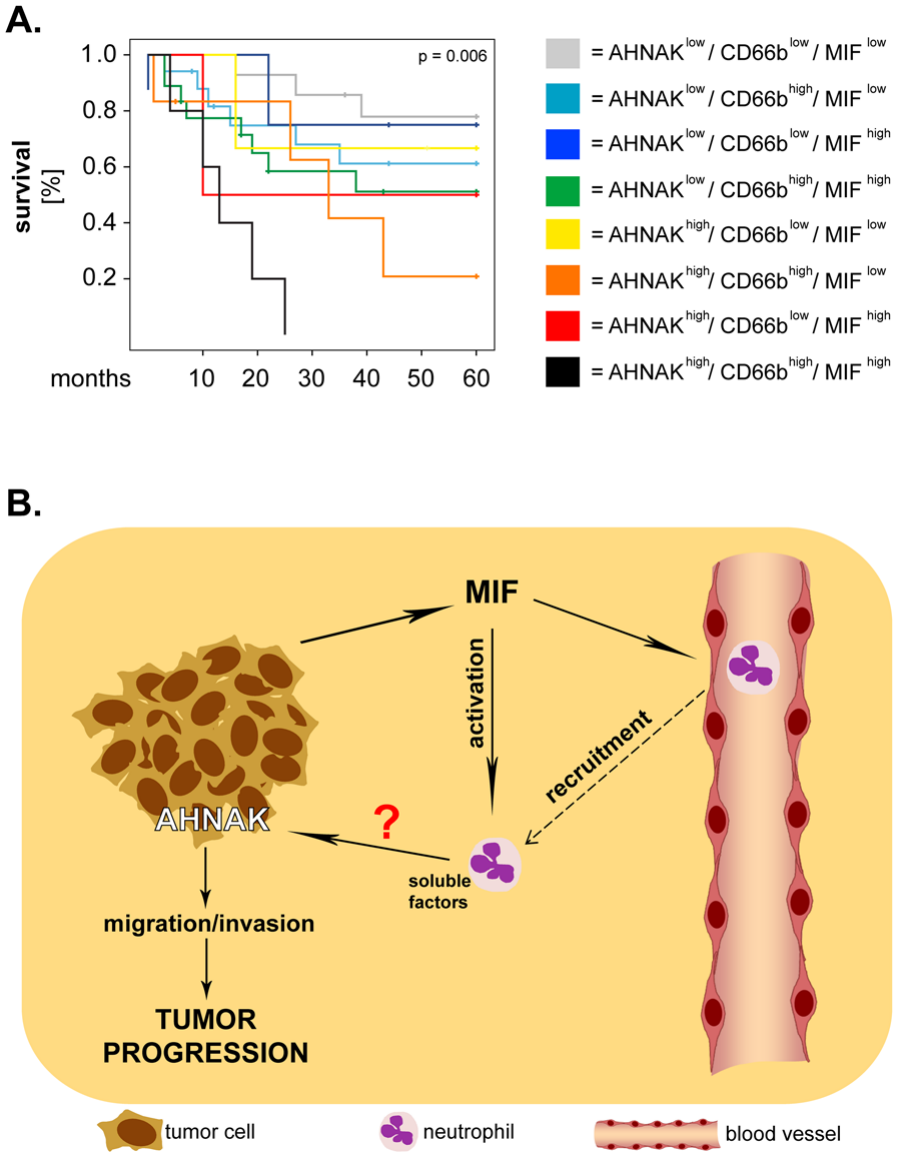
AHNAK, MIF and tumor-infiltrating neutrophils: potential interactions and relevance for the survival of larynx carcinoma patients. (**A**) Larynx carcinoma patients were divided into 8 groups corresponding to all possible combinations of AHNAK, MIF and CD66b. Survival curves were plotted according to the Kaplan-Meier method and significance was tested with the log-rank test. (**B**) Proposed model of interaction between AHNAK, MIF and neutrophils. Tumor cells release MIF which recruits and activates neutrophils, resulting in production of pro-inflammatory factors by these cells. Neutrophil-derived factors might activate AHNAK, whereby promoting tumoral migration/invasion and, ultimately, progression of larynx carcinoma.

Finally, we performed exploratory studies to determine the prognostic value of all three markers taken in combination (8 combinations) for the survival of larynx carcinoma patients. Interestingly, Kaplan-Meier analysis showed that the patients with synchronous high expression of all markers (AHNAK^high^/CD66b^high^/MIF^high^) had the shortest overall survival in our patient cohort (p = 0.006; log-rank) (**Figure 5A**). These results were further strengthened by multivariate Cox regression analysis which demonstrated that patients with the ‘triple-high' phenotype had the highest risk of death (HR = 13.80, 95% CI = 2.98–63.87, p = 0.001) compared to the other groups of patients (Table 3).

## Discussion

Increased effort has been made to identify cellular/molecular factors that could provide accurate information regarding cancer diagnosis, prognosis and response to therapy. In this study, we identified AHNAK as a novel independent prognostic factor for the overall survival of larynx carcinoma patients. Furthermore, we demonstrated that combined analysis of inflammatory markers (MIF and CD66b) with AHNAK or with one another has higher significance regarding survival than analysis of the individual markers. Thus, our study proposes novel strategies for a more accurate prognosis in larynx carcinoma and suggests potential mechanisms of tumor progression.

The role of AHNAK in cancer is poorly characterized at present. In fact, a very recent PubMed search using the keywords 'AHNAK’ or ‘desmoyokin' and 'cancer' resulted in less than 20 hits. Most of the studies on this topic were performed *in vitro*, on cancer cell lines and/or investigated AHNAK at gene rather than at protein level [11], [12], [27]. In our study, we analysed the expression levels of AHNAK by immunohistochemistry in tumor tissues from 83 larynx carcinoma patients. The results demonstrated that overexpression of tumoral AHNAK was strongly and significantly associated with poor survival of these patients, both in univariate and multivariate analysis. Thus, to the best our knowledge, we are the first to investigate AHNAK in a patient cohort of relevant size. Most importantly, our study is the first to identify AHNAK as a potential prognostic marker in cancer.

Of particular interest and novelty are also our findings regarding combination of inflammatory markers with AHNAK. In this study we tested the relevance of tumoral MIF and of tumor-infiltrating neutrophils (CD66b-positive cells) for the survival of larynx carcinoma patients. Both MIF and neutrophils have been previously linked to poor clinical outcome in orohypopharynx carcinoma patients [20], [21]. Our results showed that combined analysis of these markers with AHNAK associated strongly with poor survival in larynx carcinoma patients. Specifically, we found that patients with high AHNAK and high MIF levels (AHNAK^high^/MIF^high^) or high neutrophilic infiltration (AHNAK^high^/CD66b^high^), respectively, had significantly shorter overall survival than the other groups of patients. Furthermore, the statistical significance of these combinations was stronger than that of the highly significant AHNAK. Thus, our findings indicate that combined analysis of AHNAK with either MIF or CD66b might be a good strategy for an accurate prognosis in larynx carcinoma patients.

Additionally, these findings suggest that AHNAK might 'cooperate' with MIF and/or neutrophils to enhance progression of larynx carcinoma. A synergistic effect between cellular and molecular factors regarding tumor progression is supported by combined analysis of MIF and neutrophils in our cohort of patients. Specifically, we observed that synchronous high levels of neutrophilic infiltration and of MIF (CD66b^high^/MIF^high^) were significantly associated with poor survival, although the individual markers were less so. These findings indicate that MIF and neutrophils might interact with each other in the tumor microenvironment to enhance the progression of larynx carcinoma. Recently, our group identified direct interactions between head and neck cancer (HNC)-derived MIF and neutrophils both *in vitro* and *in situ*. We showed that HNC-derived MIF enhanced neutrophil chemotaxis *in vitro* and that tumoral MIF levels correlated with the neutrophilic infiltration in tissues from orohypopharynx carcinoma patients [21]. Since MIF is a known ligand for CXCR2-one of the major chemokine receptors on neutrophils [28], MIF-mediated recruitment might be a critical mechanism for infiltration of HNC tissues by neutrophils. Our studies further demonstrated that HNC-derived MIF stimulated neutrophils to release large amounts of pro-inflammatory factors, among which CCL4 and MMP9 [21]. Importantly, the factors released by neutrophils upon HNC stimulation enhanced tumoral migration in a feedback manner [21]. These findings are supported by an increasing number of studies showing that neutrophils enhance the motility, migration and invasion of tumor cells via-thus far not fully identified-soluble factors and molecular mechanisms (reviewed in [19], [29]). Interestingly, AHNAK was recently linked to regulation of tumoral migration/invasion, when Shankar and co-workers elegantly demonstrated that AHNAK was essential for rearrangement of the actin cytoskeleton and pseudopodia formation [11]. Based on the above-mentioned findings, it would be tempting to hypothesize that neutrophils might enhance tumoral migration/invasion via AHNAK. If this hypothesis proves correct, the progression of larynx carcinoma could be modulated by interactions between all three ‘players’ (Figure 5B). Such a model seems to be supported by our exploratory studies showing that synchronous high expression of the three markers (AHNAK^high^/CD66b^high^/MIF^high^) associates with the shortest overall survival in larynx carcinoma patients. These findings need, however, to be confirmed on larger cohorts of patients and (some) of the proposed interactions have yet to be addressed and proven experimentally in future studies.

In summary, our study identifies novel molecular and cellular factors that might serve as prognostic biomarkers and might interact with each other to enhance progression of laryngeal cancer. Ultimately, these findings contribute to a better understanding and foster the development of improved therapeutic strategies against larynx carcinoma and, perhaps, other types of solid cancer as well.
